# Biomechanical comparative analysis of effects of dynamic and rigid fusion on lumbar motion with different sagittal parameters: An *in vitro* study

**DOI:** 10.3389/fbioe.2022.943092

**Published:** 2022-08-19

**Authors:** Wei Wang, Chao Kong, Fumin Pan, Yu Wang, Xueqing Wu, Baoqing Pei, Shibao Lu

**Affiliations:** ^1^ Department of Orthopedics, Xuanwu Hospital, Capital Medical University, Beijing, China; ^2^ National Clinical Research Center for Geriatric Diseases, Beijing, China; ^3^ Beijing Key Laboratory for Design and Evaluation Technology of Advanced Implantable and Interventional Medical Devices, Beijing Advanced Innovation Center for Biomedical Engineering, School of Biological Science and Medical Engineering, Beihang University, Beijing, China

**Keywords:** Coflex interspinous stabilization, lumbar fusion, range of motion, sagittal parameter, adjacent segment degeneration

## Abstract

**Background:** Although the management of the lumbar disease is highly dependent on the severity of the patient’s condition, optimal surgical techniques to reduce the risk of adjacent degeneration disease (ADS) remain elusive. Based on *in vitro* biomechanical tests of the cadaver spine, this study aimed to comparatively analyze the kinematic responses of the spine with dynamic and rigid fixations (i.e., Coflex fixation and posterolateral fusion) after single-or double-level lumbar fusion in daily activities.

**Methods:** Six human lumbar specimens (L1-S1) were selected for this experiment, and the sagittal parameters of each lumbar specimen were measured in the 3D model. The specimens were successively reconstructed into five groups of models: intact model, single-level L4-5 Coflex fixation model, single-level L4-5 Fusion (posterior pedicle screw fixation) model, double-level L4-5 Coflex + L5-S1 Fusion model; and double-level L4-5 Fusion + L5-S1 Fusion model. The pure moment was applied to the specimen model to simulate physiological activities in daily life through a custom-built robot testing device with an optical tracking system.

**Results:** For single-level lumbar fusion, compared to the traditional Fusion fixation, the Coflex dynamic fixation mainly restricted the extension of L4-L5, partially retained the range of motion (ROM) of the L4-L5 segment, and reduced the motion compensation of the upper adjacent segment. For the double-level lumbar fixation, the ROM of adjacent segments in the Coflex + Fusion was significantly decreased compared to the Fusion + Fusion fixation, but there was no significant difference. In addition, PT was the only sagittal parameter of the preoperative lumbar associated with the ROM under extension loading. The Coflex fixation had little effect on the original sagittal alignment of the lumbar spine.

**Conclusion:** The Coflex was an effective lumbar surgical technique with a less altering kinematic motion of the lumbar both at the index segment and adjacent segments. However, when the Coflex was combined with the fusion fixation, this ability to protect adjacent segments remained elusive in slowing the accelerated degradation of adjacent segments.

## Introduction

Lumbar fusion with posterior instrumentation has been the gold standard for lumbar spine intervention treatment. Traditional lumbar fusion has intrinsic issues in some cases, such as longer operational time, higher blood loss, and greater stiffness, and may result in over-treatment of the patient. The longitudinal retrospective investigation of lumbar fusion, on the other hand, found that rigid fixation accelerated secondary degeneration of adjacent segments (Whitecloud et al., 1994; Louie et al., 2019; Wang et al., 2021). Spine fusions disrupt the mechanical environment inside the vertebral body, affect blood oxygen and nourishment delivery, and cause postoperative complications of adjacent segments after spinal fusion (Zhou et al., 2016). The high incidence of secondary accelerated degenerative diseases at adjacent levels after lumbar fusion is still a problem for orthopedic surgeons.

To overcome the limits of traditional fusion, emerging non-fusion techniques with motion preservation are designed to achieve sufficient stability and slow the degeneration process by restoring partially segmental kinematics, allowing for more physiological load transmission (Sangiorgio et al., 2011; Zhou et al., 2020; Zheng et al., 2021). A Coflex interspinous stabilization, as the third joint offloads the two facets, provides neutral equilibrium of lumbar disorders and minimizes stress concentration in adjacent segments, preventing the occurrence of ASD. Several previous studies have suggested that the Coflex system is safe and effective (Zhao et al., 2018; Shen et al., 2021). Zheng et al. (2021) compared the radiographic outcomes of patients after single-level Coflex stabilization and traditional posterior fusion for a minimum of 8 years and found no significant difference between the two groups at each time point. The superiority of that dynamic, flexible surgical stabilization over traditional fusions, however, remains elusive, especially considering that the selection of surgery is highly dependent on the severity of the patient’s condition.

The key to maintaining the static and dynamic balance of the human body is sagittal spine alignment, which minimizes the energy consumption of the trunk in daily activities. Clinical studies have suggested that sagittal balance is important in developing therapeutic strategies for a variety of spinal disorders (Ferrero et al., 2016; Sebaaly et al., 2018; Sebaaly et al., 2020). Spinopelvic radiographs have gradually become the standard in clinic for giving information on pathological diagnosis or preoperative planning (Roussouly et al., 2005; Bari et al., 2020; Sebaaly et al., 2020). Roussouly et al. (2005) proposed four types of sagittal alignment of the normal spine, which was defined by several sagittal parameters of the lumbar spine, such as pelvic incidence (PI), sacral inclination (SS), pelvic tilt (PT), lumbar lordosis (LL), etc. The optimum spinal surgery treatment should alleviate focal segmental illness and restore lumbar spine stability. Simultaneously, surgical procedures aim to minimize the impact on the overall biomechanical stability of the lumbar spine, especially in adjacent segments. Many patients with lumbar degeneration have some degree of movement instability and obstacles (Kettler et al., 2012; Pieler-Bruha, 2016). The *in vivo* and *in vitro* kinematic study of the human spine is still a challenging task. There have been few studies on assessing the effect of dynamic or rigid fixation on spinal motion on a laboratory platform considering the sagittal alignment of the spine due to a shortage of human donor cadaver spines and complicated experimental procedures.

The goal of this study was to establish an experimental assessment method for spinal biomechanics research after lumbar fusion, considering spinal kinematics and sagittal alignment. We investigated how the dynamic Coflex and traditional fusions and fixed segments (single or double-level lumbar fusion) influenced the range of motion (ROM) of the lumbar, especially on adjacent segments. This study also tried to find out whether normal sagittal parameters before fusion correlated with the ROM of the spine after different lumbar fusions. These results partially bridged the gap in understanding the biomechanical response of the spine to dynamic and traditional fixation devices and provide references for understanding the accelerated degeneration of adjacent segments and optimizing the application of spinal internal fixation.

## Materials and methods

### Specimen preparation

Approved by the Bioethics and Medical Ethics Committee, Beihang University (No.: BM20190009), Six donated human lumbar spines (L1–S1 segments, three females, three males, 32–64 years of age) were enrolled in the experimental study. The spiral computed tomography (CT) with a slice thickness of 0.6 mm (Light Speed Pro16, GE, Waukesha, WI, United States) was conducted to exclude the lumbar spines with disc degeneration, bony defects, scoliosis, tumors, a history of back surgery, or prolonged bed rest before death. Muscles around the lumbar spine were removed to gain the osteoligamentous structure, but be careful to preserve discs, facets and ligaments (Wilke et al., 1998). The specimens were partially frozen and wrapped in cling film before testing to reduce water loss.

### Parameters measurement

**FIGURE 1:**
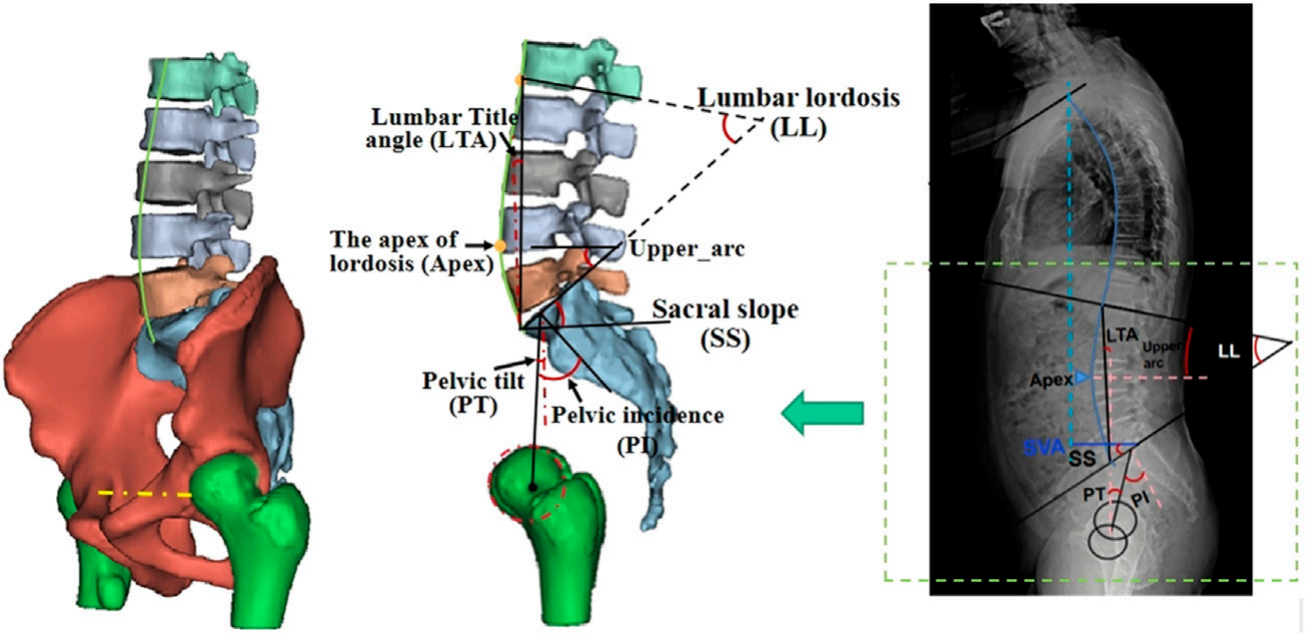
Measurement of sagittal parameters in the 3D lumbo-pelvic model.

A 3D model of the lumbar was reconstructed using CT images to measure the sagittal parameters. Duval-Beaupère et al. (1992), Roussouly et al. (2005) defined sagittal parameters. The lumbo-pelvic sagittal parameters included pelvic incidence (PI), sacral slope (SS), pelvic tilt (PT), lumbar lordosis (LL), the apex of lordosis (Apex), lumbar title angle (LTA), upper_arc, and the number of vertebrae in lordosis (NVL), as shown in Figure 1. The five observers measured each radiograph twice with 1 week between rounds.

### Construction of the lumbar fusion model

**FIGURE 2:**
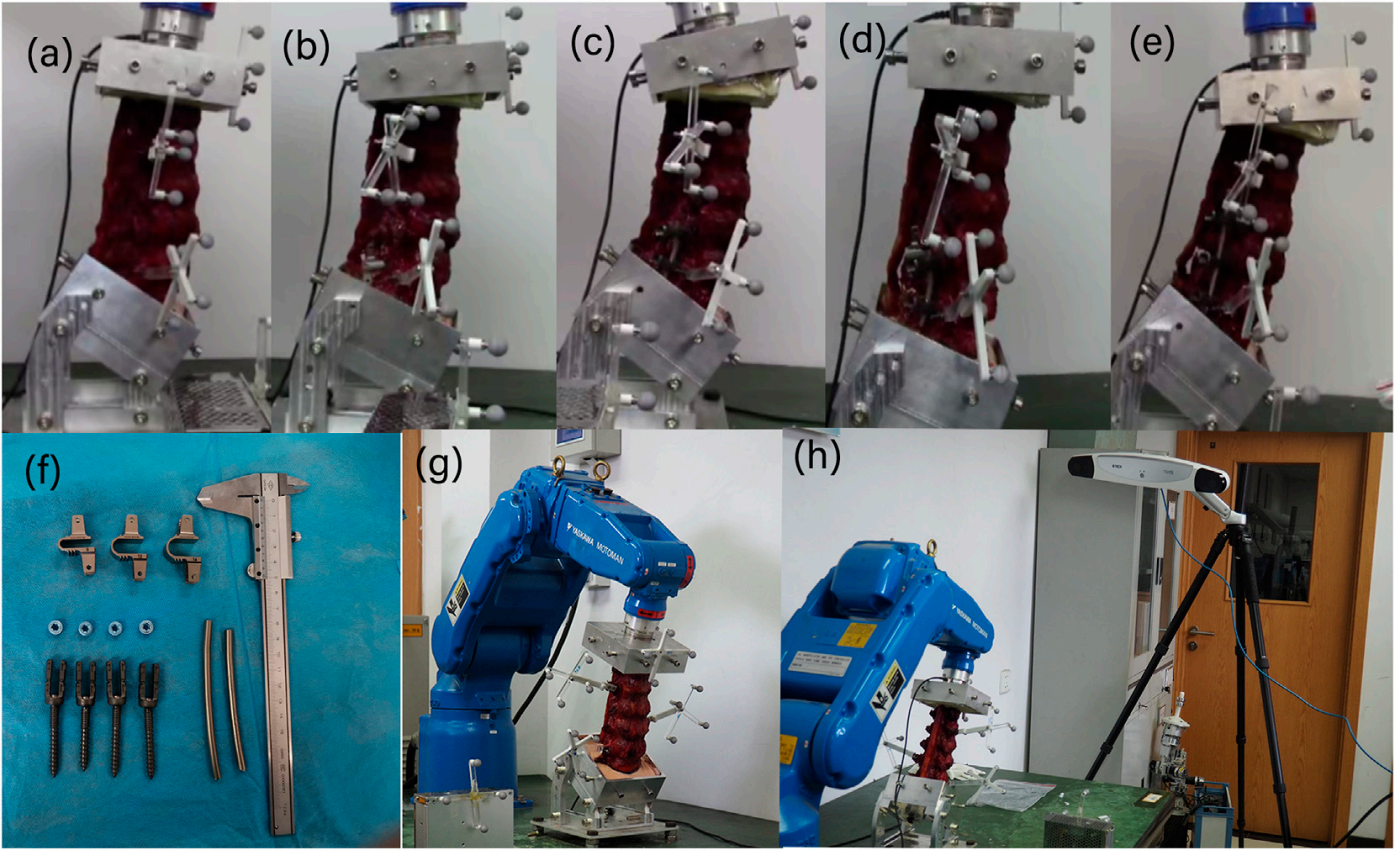
Schematic diagram of the *in vitro* experimental sample. **(A)** Normal; **(B)** L4-L5 Coflex; **(C)** L4-L5 Fusion; **(D)** Coflex + Fusion (L4-L5 Coflex + L5-S1 Fusion); **(E)** Fusion + Fusion (L4-L5 Fusion + L5-S1 Fusion). There were six samples in each group. **(F)** Implanted devices; **(G)** the robotic testing device; **(H)** 3D optoelectronic camera system.

Each specimen was successively reconstructed into five groups of models, as shown in Figure 2. 1) Normal: intact model; 2) L4-L5 Coflex: single-level L4-5 Coflex fixation model; 3) L4-L5 Fusion: single-level L4-5 pedicle screw fixation model; 4) Coflex + Fusion: double-level L4-5 Coflex + L5-S1 pedicle screw fixation model; 5) Fusion + Fusion: double-level L4-5 Coflex™ + L5-S1 pedicle screw fixation model. All the above models were made by experienced orthopedic doctors in Xuanwu Hospital.

### Testing protocol and device

A robotic testing device (NX100MH6, Kabushiki-gaisha Yasukawa Denki, Kitakyushu, Japan) published in our previous literature was performed to measure the force-displacement behavior of lumbar segments (Kong et al., 2015) (Figure 2). A force-moment sensor (Gamma, ATI Industrial Automation, Ontario, Canada) was mounted on the robot’s arm to record force and then provide feedback. Polymethylmethacrylate (PMMA) was used to fix both the L1 and S1 vertebra ends for installation in the custom-made containers (Figure 2). The 3D optoelectronic camera system (Optotrak Certus, Northern Digital Inc., Waterloo, Canada) recorded the vertebrae movement by tracking the location of five markers. The markers were attached to L1, L2, L3, L4, and L5 vertebrae, respectively.

**FIGURE 3:**
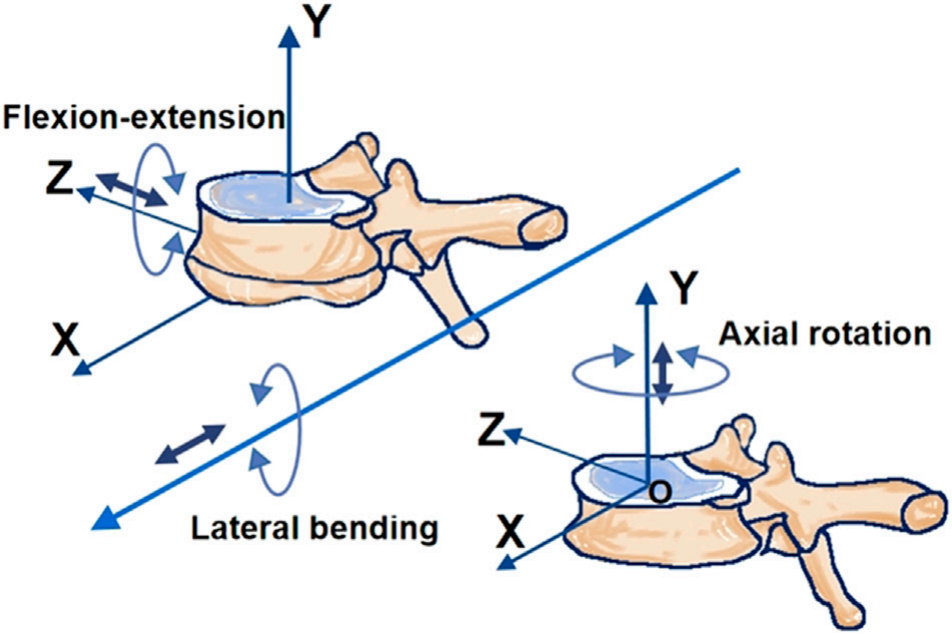
The coordinate system of two adjacent vertebral bodies.

According to the International Society of Biomechanics (ISB), the coordinating axes between adjacent vertebral bodies as shown in Figure 3. The test protocol consisted of six pure moment loads at a constant loading rate of 1.0°/s (Panjabi et al., 1992), including the flexion and extension load of 7.5 Nm, a lateral bending load of 7.5 Nm and axial rotation of 5 Nm. The robotic system automatically optimized the loading path, increasing the target load by 10% (7.5/5 Nm). In order to minimize the viscoelastic effect (Panjabi, 1998; Panjabi, 2007), the first 1.5 loading cycles were used and the following three loading cycles were recorded for analysis. During the test, the specimens were kept moist with saline (0.9%). First, Normal specimens were tested using the above method and then soaked in 0.9% saline water for 30 min. The normal specimen was implanted with the Coflex at the L4-5 segment to reconstruct the L4-5 Coflex specimen. The L4-5 Coflex specimen then repeated the above experimental steps and recovered. Similarly, L4-L5 Fusion, Coflex + Fusion, and Fusion + Fusion specimens were sequentially reconstructed and tested. In this study, the same spine specimen was reused five times. O’Connell et al. (2007) demonstrated that, in the *in vitro* tests, the original mechanical properties of the spine could be restored when soaking in a physiological saline bath for 3–4 times longer than the loading time.

### Data analysis

Data analysis was performed using SPSS software (IBM Corp, Armonk, NY, United States). Inter-rater and intra-rater reliability was assessed using intra-class correlation (ICC) coefficients. The ROM of the specimens in all the groups was measured under different loading conditions. Paired t-tests were used to compare the ROM of the same specimens in the different model groups. Spearman’s correlations were used to compare the relationships between the sagittal parameters and the ROM in the different Model groups under all loading conditions. Correlations were assumed to be strong (*r* = 0.80–1.00), moderate (*r* = 0.50–0.79), weak (*r* = 0.20–0.49), or not relevant (*r* < 0.20). p < 0.05 was considered statistically significant.

## Results

### Sagittal parameters

The sagittal parameters of the intact specimens before fusion were shown in Table 1. The average value of LL, PI, PT SS, Upper_arc and LTA was 46.88 ± 7.74°, 10.67 ± 0.92°, 36.22 ± 7.06°, 49.37 ± 6.18°, 14.90 ± 0.91°, and −4.75 ± 0.97°, respectively. The inflection point from kyphosis to lordosis almost appeared at the T12-L1 segments with an NVL of 5.01. The Apex of lumbar lordosis was located near the lower endplate L3 to the upper endplate S1. The ICC of all the parameters ranged from 0.83 to 0.97.

**TABLE 1:** Sample information.

	Sex	Age	PI (°)	PT (°)	SS (°)	LL (°)	Upper arc (°)	LTA (°)	Apex	NVL
Sample 1	Female	53	36.9	9.4	27.5	40.3	14	−5.2	Upper L5	4.3
Sample 2	Male	32	39.6	10.2	29.4	43.2	13.9	−4.4	Base L4	4.6
Sample 3	Male	55	44.2	11.4	32.8	48.2	15.4	−4.2	Base L4	4.9
Sample 4	Female	64	47.5	10.1	37.4	52.4	14.8	−5.7	Middle L4	5
Sample 5	Male	59	54.1	10.7	43.4	53.9	14.7	−5.9	Middle L4	4.8
Sample 6	Female	42	59	12.2	46.8	58.2	16.6	−3.07	Base L3	5

### Overall range of motion in a single-level lumbar fixation model

**FIGURE 4:**
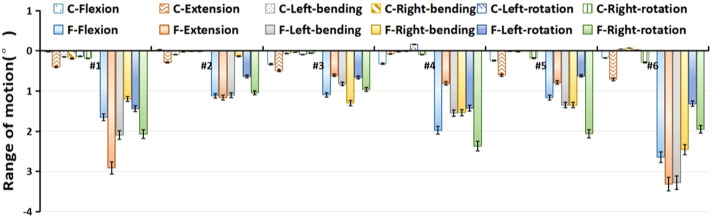
Preoperative and postoperative differences in the overall range of motion of each specimen. C: L4-L5 Coflex fixation group; F: L4-L5 Fusion fixation group.

Under different loading conditions, the overall ROM of L1-S1 segments in the two single-level lumbar fixation models (L4-L5 Coflex and L4-L5 Fusion) was shown in Figure 4. The overall ROM change in the L4-5 Coflex was minimal, essentially less than 0.3°. As shown in Table 2, the single-level Coflex fixation had a significant effect on the ROM in flexion and extension (*p* < 0.05), compared to the ROM in the Normal, but there was no significant change in the ROM in lateral bending and axial rotation loading (*p* > 0.05). For the L4-L5 Fusion model, the overall ROM decreased significantly under all loading conditions (*p* < 0.05). The ROM decreased from 1.19° to 2.91° in flexion, 0.13°–1.16° in extension, 0.60°–2.37° in lateral bending, and 0.61°–3.31° in axial rotation, respectively. The L4-L5 Fusion had a larger effect on the ROM than the L4-L5 Coflex in flexion, lateral bending, and axial rotation (*p* < 0.05). Although the decrease in ROM in the L4-L5 Fusion was larger than that in the L4-L5 Coflex under extension loading, there was no significant difference between them (*p* > 0.05).

**TABLE 2:** Significant difference in the range of motion of single-level fusion under different loading.

	Normal VS. L4-L5 Coflex	Normal VS. L4-L5 Fusion	L4-L5 Coflex VS. L4-L5 Fusion
Flexion	0.036*	0.001*	0.003*
Extension	0.006*	0.022*	0.052
Left-bending	0.152	0.005*	0.007*
Right-bending	0.388	0.007*	0.01*
Left-rotation	0.93	0.002*	0.003*
Right-rotation	0.54	0.001*	0.001*

*Significant difference *p* < 0.05.

### Intervertebral rotation distribution in a single-level fixation model

**FIGURE 5:**
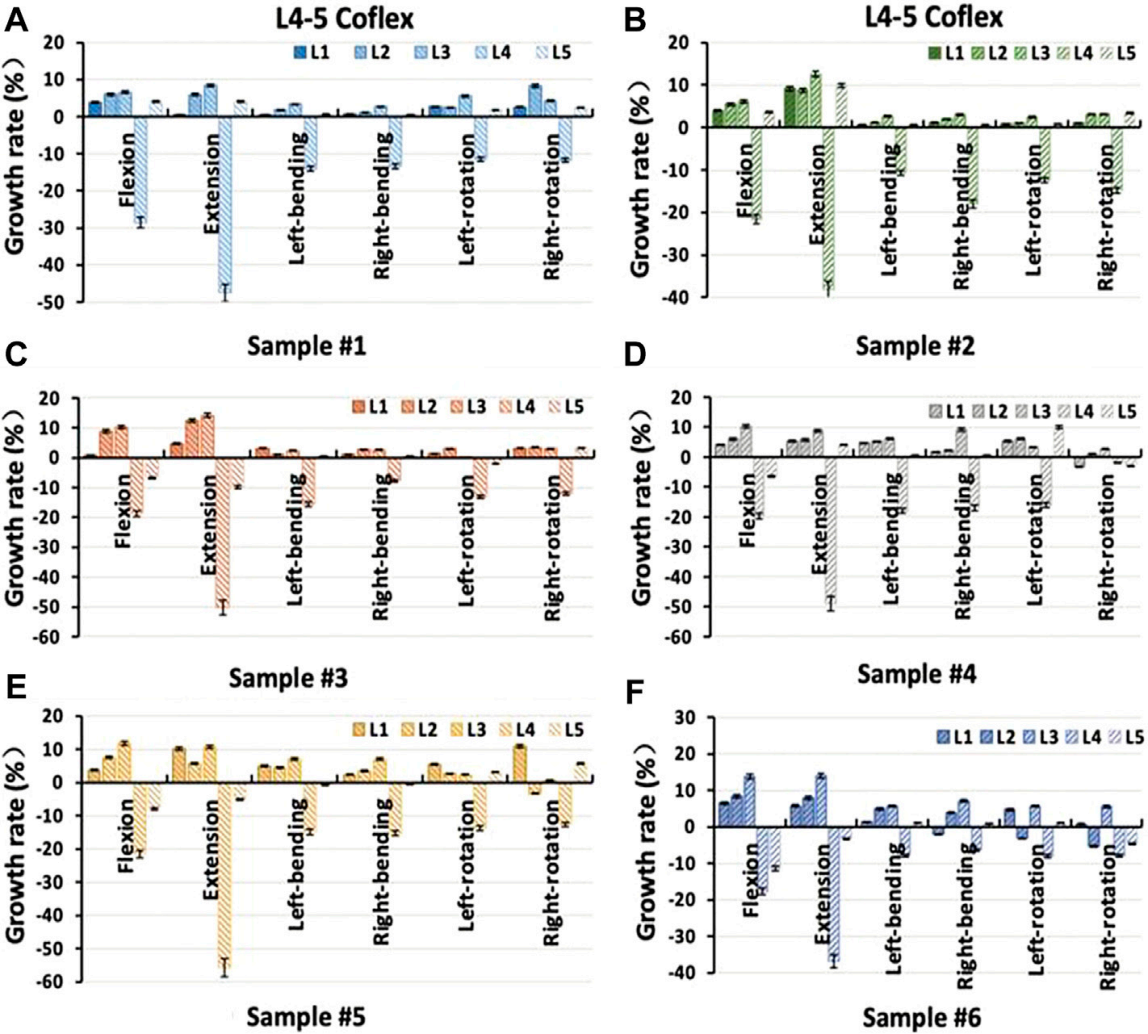
Percentage increment of the range of motion in each segment of samples after single-level L4-L5 Coflex fixation **(A)** Sample # 1; **(B)** Sample # 2; **(C)** Sample # 3; **(D)** Sample # 4; **(E)** Sample # 5 and **(F)** Sample # 6.

The distribution of the ROM of each vertebra in the L4-L5 Coflex and L4-L5 Fusion was shown in Figure 5. The Coflex dynamic fixation reduced ROM from 36.71% to 55.68% in extension, 17.73%–28.61% in flexion, and about 10% in lateral bending and axial rotation at the L4-L5 level. The increase in ROM in adjacent segments of the specimens after L4-L5 Coflex was minimal, ranging from 0.06% to 14.19%. In extension, the increase in ROM of adjacent segments was significantly greater than that under other loading conditions, indicating that the Coflex implant greatly inhibited extension movement. In the L4-L5 Fusion, the ROM of adjacent segments increased much more than in the L4-L5 Coflex, ranging from 0.05% to 27.34%.

**FIGURE 6:**
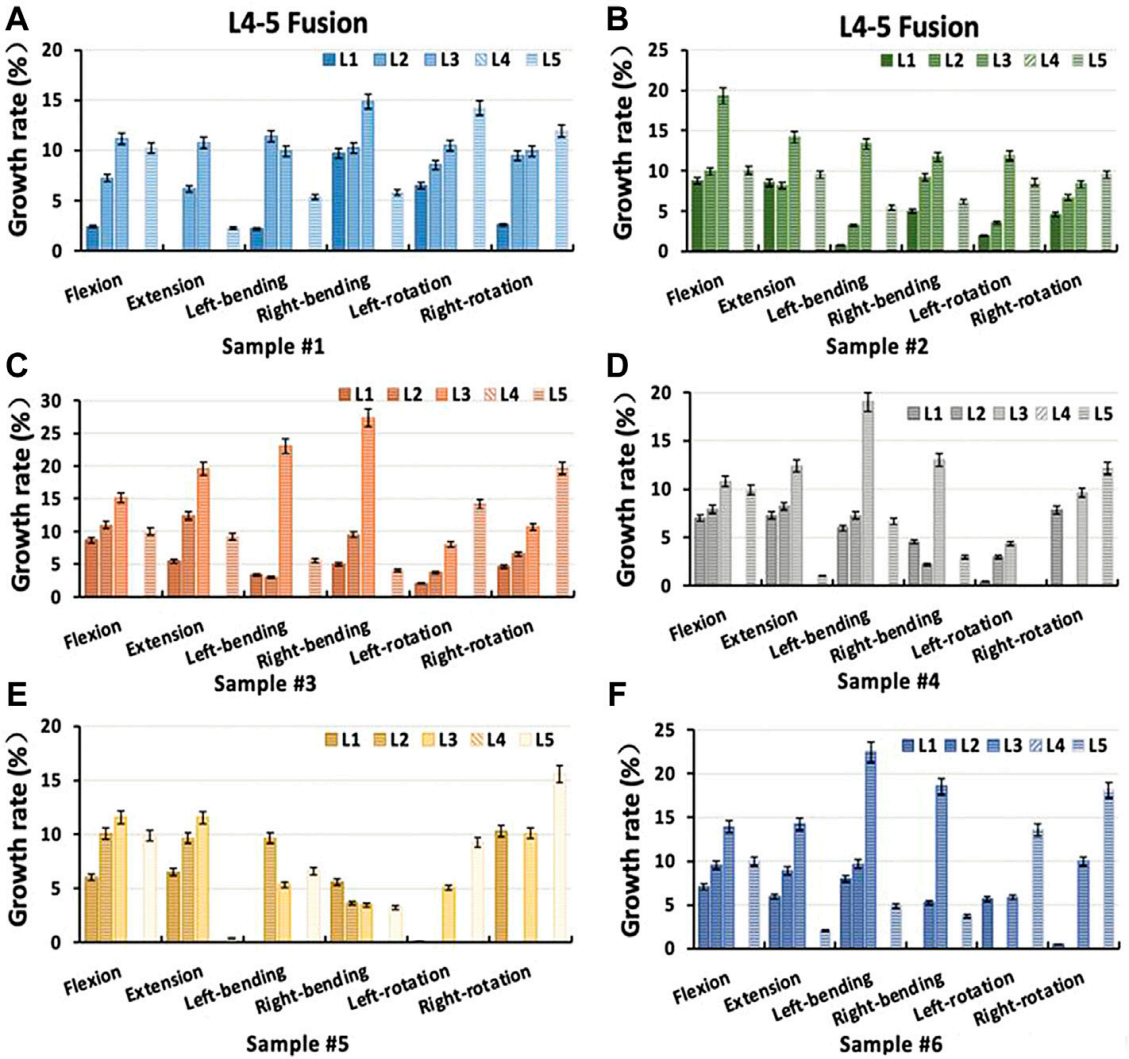
Percentage increment of the range of motion in each segment of samples after single-level L4-L5 Fusion fixation **(A)** Sample # 1; **(B)** Sample # 2; **(C)** Sample # 3; **(D)** Sample # 4; **(E)** Sample # 5 and **(F)** Sample # 6.

Under all loading conditions, the ROM significantly increased in the L4-L5 Coflex and the L4-L5 Fusion for the upper L3-L4 adjacent segment (*p* < 0.05), with an increase in the L4-L5 Fusion being much greater than that in the L4-L5 Coflex (*p* < 0.05), as shown in Figure 6 and Table 3. For the L2-L3 adjacent segments, the ROM in the L4-L5 Coflex and the L4-L5 Fusion increased significantly in flexion, extension, and lateral bending *p* < 0.05), and there was a significant difference between the two single-level fixation models (*p* < 0.05). However, for the L1-L2 adjacent segment, both single-level fixations had a significant effect on ROM in flexion and extension, with no significant difference (*p* > 0.05). Furthermore, neither the L4-L5 Coflex nor the L4-L5 Fusion had a significant effect on the ROM of the inferior L5-S1 adjacent segment.

**TABLE 3:** Significant difference in the range of motion of adjacent segments after single-level fusion under different loading.

	Normal VS. Coflex	Normal VS. Fusion	Coflex VS. Fusion		Normal VS. Coflex	Normal VS. Fusion	Coflex VS. Fusion
L3-L4				L5-S1			
Extension	0.006*	0.002*	0.017*	Extension	0.963	0.717	0.281
Left-bending	0.004*	0.007*	0.015*	Left-bending	0.939	0.398	0.004*
Right-bending	0.001*	0.002*	0.029*	Right-bending	0.051	0.55	0.001*
Left-rotation	0.023*	0.004*	0.058	Left-rotation	0.841	0.624	0.503
Right-rotation	0.015*	0.001*	0.001*	Right-rotation	0.945	0.17	0.091
L2-L3				L2-L1			
Flexion	0.001*	0.001*	0.008*	Flexion	0.009*	0.003*	0.064
Extension	0.004*	0.003*	0.116	Extension	0.014*	0.013*	0.75
Left-bending	0.019*	0.006*	0.016*	Left-bending	0.12	0.121	0.626
Right-bending	0.001*	0.001*	0.045*	Right-bending	0.11	0.068	0.034*
Left-rotation	0.334	0.375	0.754	Left-rotation	0.23	0.062	0.833
Right-rotation	0.78	0.843	0.957	Right-rotation	0.162	0.007*	0.204

*Significant difference *p* < 0.05.

### Range of motion in a two-level lumbar fixation model

**FIGURE 7:**
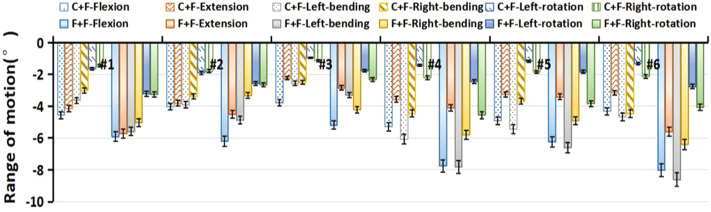
Preoperative and postoperative differences in the overall range of motion of each sample. C+F: L4-L5 Coflex + L5-S1 Fusion fixation group; F+F: L4-L5 Fusion + L5-S1 Fusion fixation group.

Under different loading conditions, the overall ROM of L1-S1 segments in the double-level lumbar fixation models (L4-L5 Coflex and L4-L5 Fusion) was shown in Figure 7. Both the Coflex + Fusion and Fusion + Fusion showed no significant influence on the ROM of the inferior L5-S1 adjacent segment (*p* < 0.05), with the former having less effect in flexion, lateral bending, and axial rotation than the latter (*p* < 0.05) (Table 4). The ROM differences between the two fixation models were as follows: 1.31°–3.68° in flexion, 0.36°–2.45° in extension, 0.29°–3.93° in lateral bending, and 0.65°–1.99° in axial rotation.

**TABLE 4:** Significant difference in the range of motion of two-level fusion under different loading.

	Normal VS. Coflex + Fusion	Normal VS. Fusion + Fusion	Coflex + Fusion VS. Fusion + Fusion
Flexion	0.002*	0.001*	0.007*
Extension	0.001*	0.001*	0.001*
Left-bending	0.004*	0.001*	0.002*
Right-bending	0.015*	0.002*	0.004*
Left-rotation	0.004*	0.002*	0.009*
Right-rotation	0.013*	0.006*	0.013*

*Significant difference *p* < 0.05.

### 3.5 Intervertebral rotation distribution in a two-level fixation model

**FIGURE 8:**
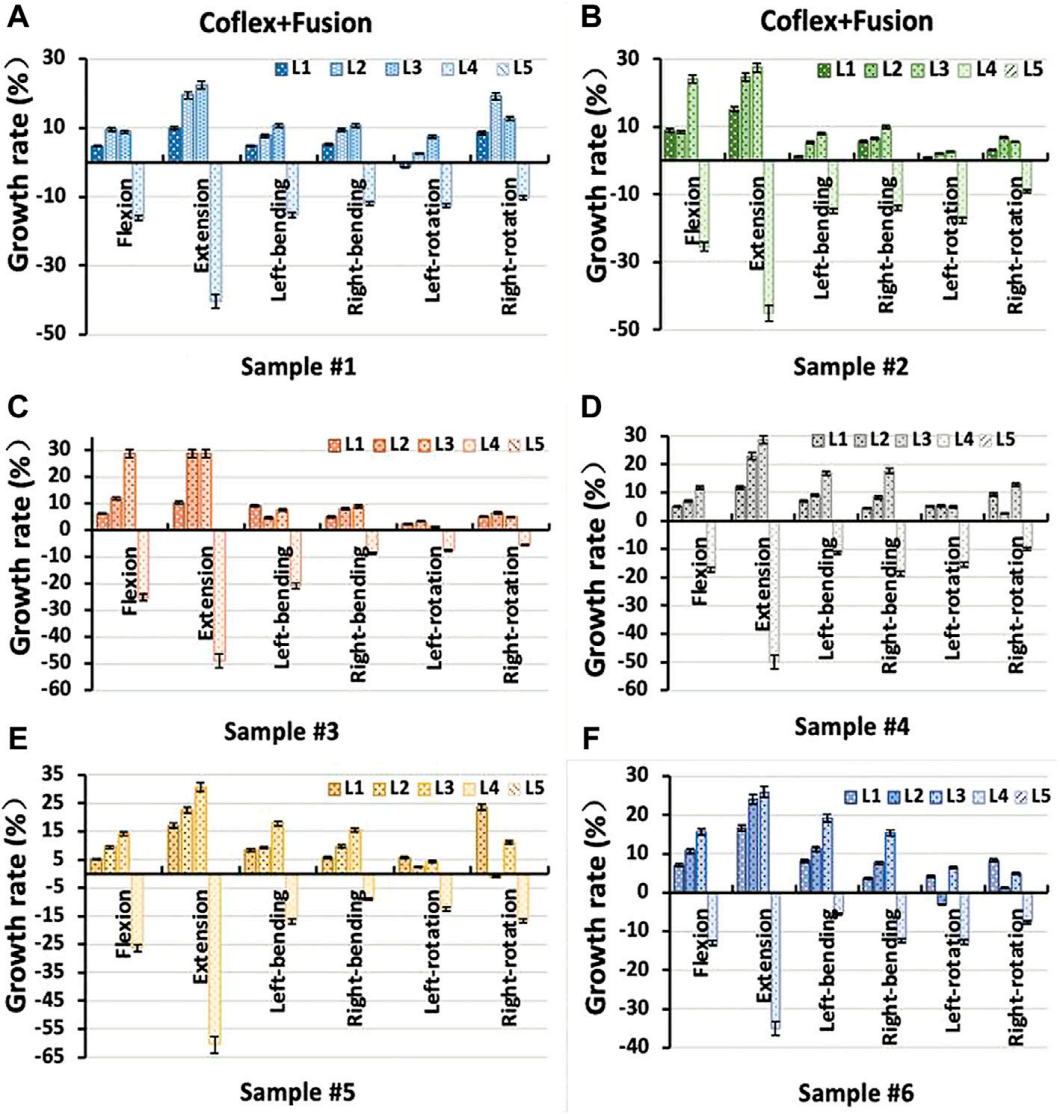
Percentage increment of the range of motion in each segment of samples after two-level Coflex + Fusion fixation **(A)** Sample # 1; **(B)** Sample # 2; **(C)** Sample # 3; **(D)** Sample # 4; **(E)** Sample # 5 and **(F)** Sample # 6.

The Coflex dynamic fixation maintained the partial ROM at the L4-L5 level, with a decrease in extension of 40.28%–60.01%, in flexion of 13.09%–26.30%, in lateral bending of 5.48%–20.89%, and in axial rotation of 5.54%–17.59% (Figure 8). In the Coflex + Fusion, the ROM of adjacent segments increased by 4.79%–28.88% in flexion, 1.28%–19.25% in lateral bend, and −3.08% to 12.80% in axial rotation, respectively. In extension, the increase in ROM of adjacent segments was significantly larger than that in other loading conditions, ranging from 9.96% to 30.60%. In the Fusion + Fusion, the ROM of adjacent segments increased by 10.57%–36.17% in flexion, 5.92%–32.40% in extension, 4.78%–29.31% in lateral bend, and −3.12% to 24.70% in axial rotation, respectively. The ROM of adjacent segments in the Fusion + Fusion was significantly larger than that of adjacent segments in the Coflex + Fusion (*p* < 0.05).

**FIGURE 9:**
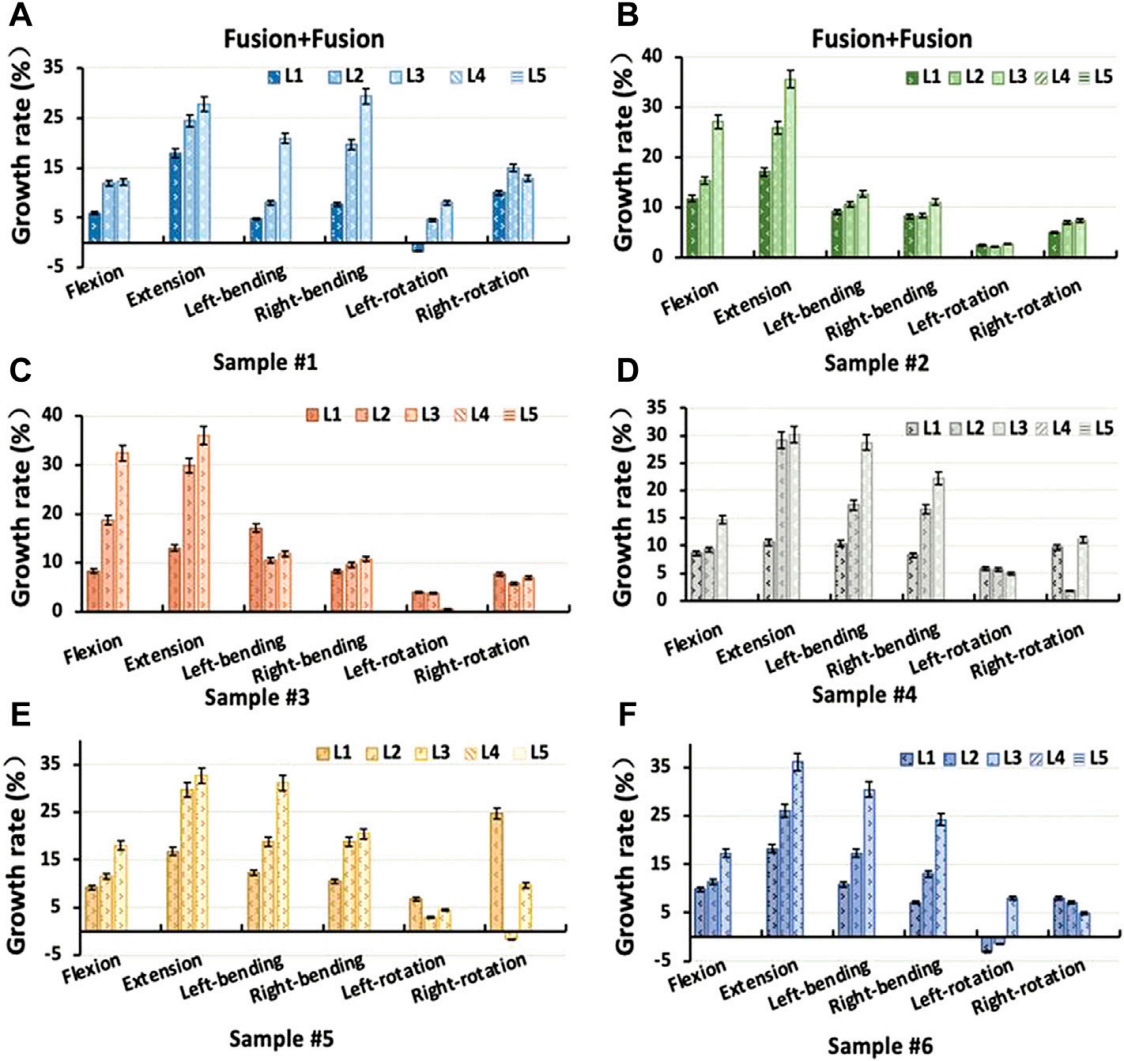
Percentage increment of the range of motion in each segment of samples after two-level Fusion + Fusion fixation **(A)** Sample # 1; **(B)** Sample # 2; **(C)** Sample # 3; **(D)** Sample # 4; **(E)** Sample # 5 and **(F)** Sample # 6.

For the upper L3-L4 adjacent segment, the ROM significantly increased in the Coflex + Fusion and the Fusion + Fusion under all loading conditions (*p* < 0.05), while there was a significant difference only in flexion and lateral bending (*p* < 0.05), as shown in Figure 9 and Table 5. Similarly, for the L1-L2 and L2-L3 adjacent segments, the double-level fixation had a significant effect on the ROM in flexion, extension and lateral bending, with a significant difference only in flexion and lateral bending (*p* > 0.05).

**TABLE 5:** Significant difference in the range of motion of adjacent segments after single-level fusion under different loading.

	Normal VS. C+F	Normal VS. F+F	C+F VS. F+F		Normal VS. C+F	Normal VS. F+F	C+F VS. F+F
L3-L4				L2-L3			
Flexion	0.001*	0.001*	0.001*	Flexion	0.001*	0.001*	0.017*
Extension	0.001*	0.003*	0.052	Extension	0.002*	0.002*	0.066
Left-bending	0.005*	0.005*	0.006*	Left-bending	0.003*	0.003*	0.01*
Right-bending	0.001*	0.003*	0.044*	Right-bending	0.001*	0.002*	0.011*
Left-rotation	0.013*	0.02*	0.32	Left-rotation	0.282	0.102	0.085
Right-rotation	0.003*	0.001*	0.767	Right-rotation	0.061	0.053	0.611
L2-L1							
Flexion	0.002*	0.001*	0.003*	Extension	0.007*	0.054*	0.113
Left-bending	0.005*	0.001*	0.021*	Right-bending	0.001*	0.001*	0.081
Left-rotation	0.055	0.861	0.389	Right-rotation	0.051	0.071	0.082

*Significant difference *p* < 0.05; C+F: L4-L5 Coflex + L5-S1 Fusion fixation group; F+F: L4-L5 Fusion + L5-S1 Fusion fixation group.

### Correlation analysis

The correlation between the original sagittal parameters of the lumbar-pelvis and the ROM in the different fixation models under different loading conditions was shown in Table 6. Only in the L4-L5 Coflex in flexion, the ROM was correlated with four parameters, including PI (*r* = 0.943), SS (*r* = 0.943), LL (*r* = 0.943) and Apex (*r* = −0.883). In extension, PT had a strong correlation with the ROM both in the single- and double-level fusions. There was no correlation between other sagittal parameters of the normal specimens and the ROM under all loading conditions.

**TABLE 6:** Correlation between the lumbar-pelvic parameter and the range of motion after fusion under different loading.

Fusion	Loading	PI	PT	SS	LL	Apex	Upper arc	LTA	NVL
L4-L5 Coflex	Flexion	0.943*	0.600	0.943*	0.943*	−0.883*	0.771	0.029	0.725
	Extension	0.371	0.886*	0.371	0.371	−0.294	0.543	0.714	0.406
	Left-bending	0.600	−0.086	0.600	0.600	−0.618	0.086	−0.657	0.290
	Right-bending	0.600	−0.086	0.600	0.600	−0.706	0.086	−0.543	0.493
	Left-rotation	0.143	−0.143	0.143	0.143	−0.265	0.143	0.257	0.145
	Right-rotation	−0.14	0.143	−0.143	−0.143	0.088	0.143	0.771	−0.087
L4-L5 Fusion	Flexion	−0.086	0.543	−0.086	−0.086	0.118	−0.086	0.600	−0.174
	Extension	0.371	0.886*	0.371	0.371	−0.294	0.543	0.714	0.406
	Left-bending	0.200	0.543	0.200	0.200	−0.206	0.714	0.771	0.638
	Right-bending	−0.029	0.371	−0.029	−0.029	0.088	0.657	0.771	0.319
	Left-rotation	−0.714	−0.143	−0.714	−0.714	0.706	−0.543	0.429	−0.725
	Right-rotation	0.371	0.600	0.371	0.371	−0.147	0.600	0.143	0.203
Coflex + Fusion	Flexion	0.143	0.714	0.143	0.143	−0.088	0.257	0.600	0.203
	Extension	0.771	0.829*	0.771	0.771	−0.736	0.943*	0.543	0.899*
	Left-bending	0.771	0.257	0.771	0.771	−0.794	0.371	−0.143	0.435
	Right-bending	0.543	−0.200	0.543	0.543	−0.618	0.143	−0.600	0.435
	Left-rotation	0.029	−0.371	0.029	0.029	−0.088	0.029	−0.086	−0.058
	Right-rotation	−0.200	−0.829*	−0.200	−0.200	0.088	−0.429	−0.771	−0.145
Fusion + Fusion	Flexion	0.086	0.600	0.086	0.086	0.000	0.086	0.314	0.058
	Extension	0.543	0.943*	0.543	0.543	−0.500	0.600	0.771	0.493
	Left-bending	0.714	0.143	0.714	0.714	−0.706	0.200	−0.429	0.290
	Right-bending	0.029	−0.371	0.029	0.029	−0.088	0.029	−0.086	−0.058
	Left-rotation	0.174	−0.232	0.174	0.174	−0.239	0.145	0.000	0.074
	Right-rotation	−0.543	−0.943*	−0.543	−0.543	0.500	−0.600	−0.771	−0.493

*Significant difference *p* < 0.05. PI: pelvic incidence; PT, pelvic tilt; SS, sacral slope; LL, lumbar lordosis; Apex, the apex of lordosis; LTA, lumbar title angle; NVL, the number of vertebrae in lordosis.

## Discussion

In this study, we evaluated the kinetic response of the lumbar after different fusion techniques and fixed segments, especially adjacent segments, in combination with *in vitro* biomechanical testing and spinopelvic radiographic parameters. Both single or double-level spinal fusion had the greatest effect on the ROM of the lumbar under flexion loading, followed by lateral bending, extension and axial rotation loading. The upper adjacent segment was the most influenced by the implant in all fusion models, with the most significant compensatory movement, while the effect diminished as the distance between the adjacent segments increased. The implant, it was thought, altered the geometry of the spine and reconstructed the sagittal parameters match. As a result, neither after single-level lumbar fusion nor after double-level lumbar fusion, most sagittal parameters in the normal spine before fusion correlated with the ROM of the spine.

The Coflex dynamic implantation for single-level fixation remained partial movement of the target segment, affecting only the range of movement under extension loading. These findings were consistent with the previous research. Wilke et al. (1998) demonstrated that Coflex dynamic fixation reduced the ROM in the posterior extension of the lumbar spine by approximately 50% compared to the intact lumbar spine, and the ROM was not significantly affected in flexion, lateral bending, and rotation. Pan et al. (2016) also found no significant effects on adjacent segments in the lumbar model after Coflex fixation. The Fusion fixation had a greater effect on the ROM than that of the Coflex fixation, but both limited the extension movement. The ROM of the upper L3-l4 adjacent segment was affected by both fusion methods, however, there was no significant effect on the ROM of the inferior L5-S1 adjacent segment. The result was consistent with the clinical cases, that upper adjacent segments were more prone to secondary accelerated degeneration.

For double-segment internal fixation, although Coflex + Fusion fixation had less effect on the motion of adjacent segments than that of Fusion + Fusion fixation, the ROM of adjacent segments also significantly increased. Similar results were reported by Mageswaran et al. (2012) that the ROM of adjacent segments in an *in vitro* experimental model significantly increased in flexion, extension, and axial rotation after L3-L4 semi-rigid screw dynamic fixation + L4-L5 fusion fixation. Strube et al. (2010) also showed that, after dynamic fixation combined with fusion fixation, the ROM of the upper adjacent segments still increased significantly. It was worth noting that the protection of adjacent segments by this dynamic fixation method may not delay the degeneration of adjacent segments in the case of double-segment fixation.

Sagittal alignment plays a critical role in the biomechanical adaptation and compensation of the spine. Our previous study suggested that sagittal parameters were mainly correlated to the ROM response of the lumbar spine under sagittal (flexion and extension) loading, but had little effect on the ROM under lateral flexion and axial rotation loads. In this study, the preoperative lumbar PT was the only sagittal parameter associated with the overall ROM after single or double-level spinal fusions under extension loading. Roussouly and Nnadi (2010), Roussouly and Pinheiro-Franco (2011) advised that PT reflected the ability of the pelvis to rotate around the femoral head. Our results found that most sagittal parameters of the original lumbar before fusion did not correlated with the ROM after both single or double-level lumbar fusion. Traditional internal fixation with stiffness higher than vertebrae completely alters the original structure and shape of the lumbar, putting patients at risk of overtreatment. For the Coflex dynamic fixation, the ROM after fusion was still associated with PI, SS, LL, and Apex in flexion. That indicated that Coflex dynamic fixation had less interference with the original morphology of the lumbar spine, but, in the Coflex + Fusion fixation, such retention of the original morphology disappeared.

There were several limitations to the current study. Firstly, the number of samples in this study was limited due to the difficulty in obtaining qualified lumbar spine specimens. These data did not support a correlation analysis between the lumbar sagittal classification of lumbar vertebrae and ROM. Secondly, *in vitro* testing protocols and facilities for similar studies are complex and diverse. Our data cannot be directly compared with published results from other experiments. Thirdly, muscles and other soft tissues of the spine were not considered in this biomechanical testing. The results are somewhat different from the real state of the human lumbar spine. Despite these limitations, our study can provide insights into how single or double-level spinal fusion affects lumbar motion and a better understanding of the correlation between preoperative sagittal parameters and lumbar movements after different lumbar internal fixation techniques.

## Conclusion

Our findings revealed the different kinetic characteristics of the dynamic Coflex and rigid fusion devices for single and double-level lumbar fusion. The Coflex exhibited its advantage in single-level lumbar fusion that preserved partial movement of the target segment and lowered motion compensation in the upper adjacent segment. For the double-level lumbar fixation, although the range of motion of adjacent segments in the Coflex + Fusion fixation was smaller than that in the Fusion + Fusion fixation, there was no significant difference. PT was the only preoperative lumbar sagittal parameter associated with the range of motion after single and double-level fusions in extension. Coflex dynamic fixation showed the ability to reduce interference in the lumbar spine’s original shape. This study proposed a preliminary experimental assessment approach for studying the effects of various surgical implants on the biomechanical response of patients with a range of preoperative lumbar sagittal parameters.

## Data Availability

The original contributions presented in the study are included in the article/supplementary material, further inquiries can be directed to the corresponding authors.
